# Neutrophil-to-lymphocyte ratio as a predictor of mortality in patients with acute kidney injury: a systematic review and meta-analysis

**DOI:** 10.31744/einstein_journal/2025RW1555

**Published:** 2025-10-13

**Authors:** Idrys Henrique Leite Guedes, Fernando Uvinha, Isabela Lino Costa, Gustavo Martins de Ferreira, Luciano César Pontes de Azevedo, Paulo Ricardo Gessolo Lins

**Affiliations:** 1 Universidade Federal de Campina Grande Campina Grande PB Brazil Universidade Federal de Campina Grande, Campina Grande, PB, Brazil.; 2 Universidade Estadual de Campinas Campinas SP Brazil Universidade Estadual de Campinas, Campinas, SP, Brazil.; 3 Universidade Federal do Triângulo Mineiro Uberaba MG Brazil Universidade Federal do Triângulo Mineiro, Uberaba, MG, Brazil.; 4 Universidade Estadual Paulista São Paulo SP Brazil Universidade Estadual Paulista, São Paulo, SP, Brazil.; 5 Hospital Israelita Albert Einstein São Paulo SP Brazil Hospital Israelita Albert Einstein, São Paulo, SP, Brazil.; 6 Brazilian Research in Intensive Care Network (BRICNet) São Paulo SP Brazil Brazilian Research in Intensive Care Network (BRICNet), São Paulo, SP, Brazil.; 7 Universidade Federal de São Paulo Escola Paulista de Medicina São Paulo SP Brazil Escola Paulista de Medicina, Universidade Federal de São Paulo, São Paulo, SP, Brazil.

**Keywords:** Neutrohils, Injury kidney acute, Prognosis, Sensitivity and specificity

## Abstract

**Introduction:**

Acute kidney injury is a common and serious condition in critically ill patients. Current biomarkers, such as serum creatinine, are limited in early detection and prognostic assessment. The neutrophil-to-lymphocyte ratio has emerged as a promising inflammatory biomarker, reflecting systemic inflammation and potentially serving as a tool for risk stratification in high-risk groups.

**Objective:**

To evaluate the prognostic value of the neutrophil-to-lymphocyte ratio in predicting mortality in patients with acute kidney injury through a systematic review and meta-analysis.

**Methods:**

A comprehensive search was conducted in PubMed, Embase, Cochrane, and LILACS databases for full-text articles published from the inception of each database until June 2024, using terms related to neutrophil-to-lymphocyte ratio and acute kidney injury. Studies were included if they evaluated the relationship between neutrophil-to-lymphocyte ratio and mortality in acute kidney injury patients. Data were pooled for meta-analysis using a random-effects model, with sensitivity, specificity, and area under the curve (AUC) calculated.

**Results:**

Of the 806 studies identified, five met the inclusion criteria, encompassing a total of 2,424 patients. The pooled sensitivity and specificity of the neutrophil-to-lymphocyte ratio for predicting mortality were 0.58 (95%CI=0.51–0.65) and 0.65 (95%CI=0.59–0.71), respectively. The AUC was 0.65, suggesting moderate predictive power. Significant heterogeneity was observed across studies owing to differences in neutrophil-to-lymphocyte ratio cutoff points, patient populations, and outcomes analyzed.

**Conclusion:**

An elevated neutrophil-to-lymphocyte ratio is associated with a worse prognosis in patients with acute kidney injury, highlighting its potential as a readily available biomarker for risk stratification.

Prospero database registration: CRD42024569162.

## INTRODUCTION

Acute kidney injury (AKI) is one of the most prevalent and serious complications in hospitalized patients, especially in intensive care units (ICUs). This condition is characterized by an abrupt reduction in renal function, leading to the retention of nitrogenous products and hydroelectrolytic and acid-base imbalances. Acute kidney injury is associated with unfavorable outcomes, including increased mortality, prolonged hospital stays, and the need for renal replacement therapy (RRT).^(1,2)^ However, its early detection and prognosis remain challenging, partly because of the reliance on traditional markers, such as serum creatinine and urine output, which have limitations in both sensitivity and specificity for predicting the severity and progression of kidney injury.^(1,3,4)^

Creatinine, which is commonly used to assess renal function, increases only after substantial GFR loss and is influenced by factors, such as age, sex, muscle mass, and nutrition, limiting early AKI detection in certain cases. Similarly, urinary output, although frequently monitored, is affected by variables such as volume status and diuretic use, thereby reducing its reliability as a sole prognostic marker.^(4)^ These limitations highlight the need for additional biomarkers to improve AKI prognosis.

The neutrophil-to-lymphocyte ratio (NLR) has emerged as a potential inflammatory and prognostic biomarker for several clinical conditions, including cardiovascular diseases, kidney and non-kidney diabetes-related complications, cancer, AKI after contrast infusion, and systemic inflammatory disorders.^(5-8)^ The NLR, which is calculated as the ratio between the absolute number of neutrophils and lymphocytes, reflects the systemic inflammatory state, a factor implicated in the pathogenesis and progression of multiple acute and chronic conditions, and offers some advantages over traditional biomarkers, such as C-reactive protein (CRP) and interleukin-6 (IL-6), as it can be easily obtained from a complete blood count. Studies have suggested that inflammation plays a central role in the development and progression of AKI, particularly in critically ill patients, where exacerbated inflammatory responses contribute to renal tissue damage.^(9,10)^

Excessive inflammatory responses due to factors such as sepsis, major surgeries, and nephrotoxic drugs can lead to endothelial dysfunction and acute tubular injury, thereby increasing the risk of AKI progression. In this scenario, the NLR emerges as a promising, low-cost, and easily accessible marker from routine blood tests, potentially useful in everyday clinical practice, especially in resource-limited settings, to indicate the severity of AKI and its outcomes, such as mortality and the need for RRT.^(11,12)^ A prospective observational study conducted by the National Center for Health Statistics (NCHS) in the United States between 1999 and 2014 evaluated 32,454 patients. Among patients with renal disease (5,391), a strong association was found between increased NLR and mortality, with a hazard ratio (HR) of 1.62 and a 95% confidence interval (95%CI) of 1.21–2.17.^(13)^ Although studies on NLR as a prognostic biomarker show promise, variability in methodologies, populations, cutoff points, and outcome measures limits its clinical applicability.

## OBJECTIVE

Considering the findings of the above study, this systematic review and meta-analysis aimed to clarify neutrophil-to-lymphocyte ratios prognostic value in acute kidney injury-related mortality, offering insights to support more effective and personalized management strategies for patients with acute kidney injury.

## METHODS

This meta-analysis was conducted according to the PRISMA 2020 guidelines (Preferred Reporting Items for Systematic Reviews and Meta-analyses)^(14)^ and the Cochrane Handbook for Systematic Reviews of Diagnostic Test Accuracy.^(15)^ The search process included the PubMed, Embase, Cochrane and LILACS databases, using terms such as [("neutrophil-to-lymphocyte ratio" OR "neutrophil to T-lymphocyte" OR "neutrophil to lymphocyte ratio" OR "neutrophil lymphocyte ratio") AND "kidney injury"]. All relevant full-text articles published from the inception of each database until June 4, 2024, were considered, provided they were available in English (Table 1S, Supplementary Material).

The eligibility criteria included studies on adult patients with AKI that examined the relationship between high and low NLRs and 30-day mortality rates. Studies were excluded if they were duplicates, focused on mortality beyond 90 days, or lacked sufficient data for 2 × 2 table construction. When possible, 30-day mortality was prioritized; if unavailable, in-hospital mortality was used, followed by 90-day mortality. The first two authors independently selected articles meeting these criteria using the Rayyan platform and the final inclusion was determined through discussion. Data extraction enabled the calculation of combined sensitivity and specificity using 2 × 2 tables. For studies with missing or incomplete data, the corresponding authors were contacted twice over 2 weeks; lack of response resulted in the exclusion of the study owing to insufficient clinical information. To assess the quality of published studies, we use the Quality Assessment of Diagnostic Accuracy Studies-2 (QUADAS-2) tool,^(16)^ a useful and specific tool for diagnostic test studies that helps identify biases related to patient selection, test performance, reference standard use, and study timing. Heterogeneity was evaluated using the Baujat plot.^(17)^ Because the number of studies included were small, we were unable to conduct a comprehensive assessment of publication bias. Both the Egger test and funnel plot yield limited results when the meta-analysis includes fewer than 10 studies.^(18,19)^

Preprints, conference abstracts, and grey literature were excluded, and statistical analyses were conducted in R Studio using the "meta"^(20)^ and "metaprop" packages with a random effects model to account for study heterogeneity.^(21)^

## RESULTS

**Figure 1:**
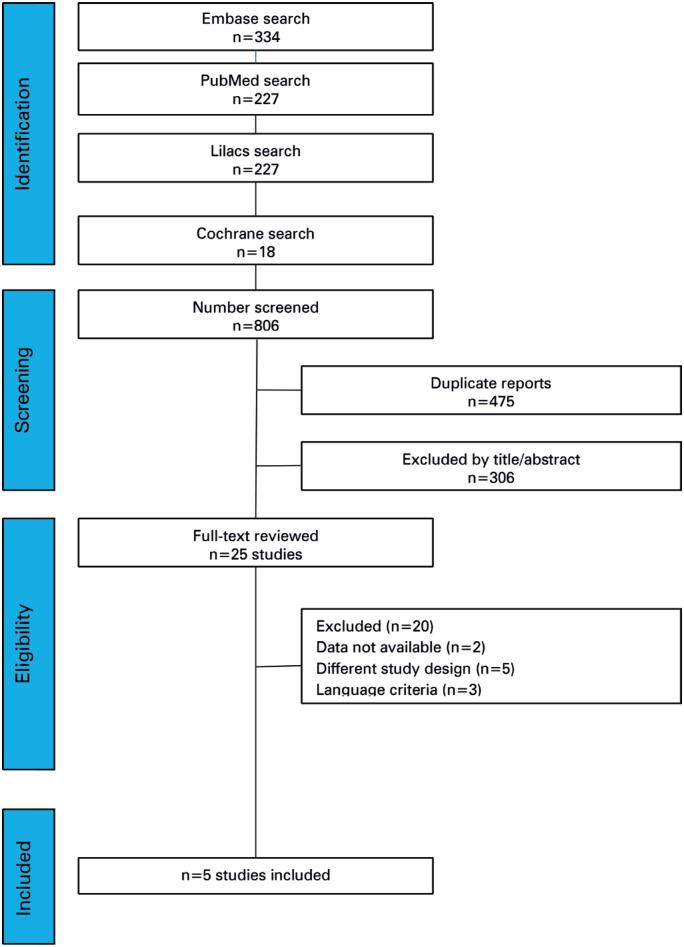
PRISMA screening diagram

The initial search identified 806 studies, of which 475 were excluded because of duplication. Following abstract review, 25 studies met the eligibility criteria for full-text assessment. Of these, 3 studies were excluded because they were not written in English, 12 lacked sufficient data to construct a 2 × 2 sensitivity table, and 5 had a different study design that did not meet the inclusion criteria. Consequently, only five studies were included in the final meta-analysis (Figure 1).

These studies included 2,424 patients, with a mean age of 59.5 years. Some studies have evaluated the performance of NLR in specific groups of patients, such as those with liver cirrhosis^(22)^ and sepsis.^(23)^ Detailed characteristics of the studies are presented in table 1.

**Table 1:** Baseline characteristics of studies

Study	Year	Country	Study design	Patients	Mean age (years)	SD	Cut-off NLR	Mortality (days)
Musunuri et al.^(22)^	2023	India	Prospective	102	53.2	5.94	5.46	90
Wei et al.^(23)^	2024	China	Retrospective	413	63	17	1.71	30
Lee et al.^(24)^	2024	South Korea	Retrospective	798	64.2	14.5	18	In hospital
Chen et al.^(25)^	2021	China	Retrospective	802	NA	NA	9	90
Wei et al.^(26)^	2023	China	Retrospective	309	57.8	18.1	12.12	30

SD standard deviation; NLR neutrophil-to-lymphocyte ratio.

**Figure 2:**
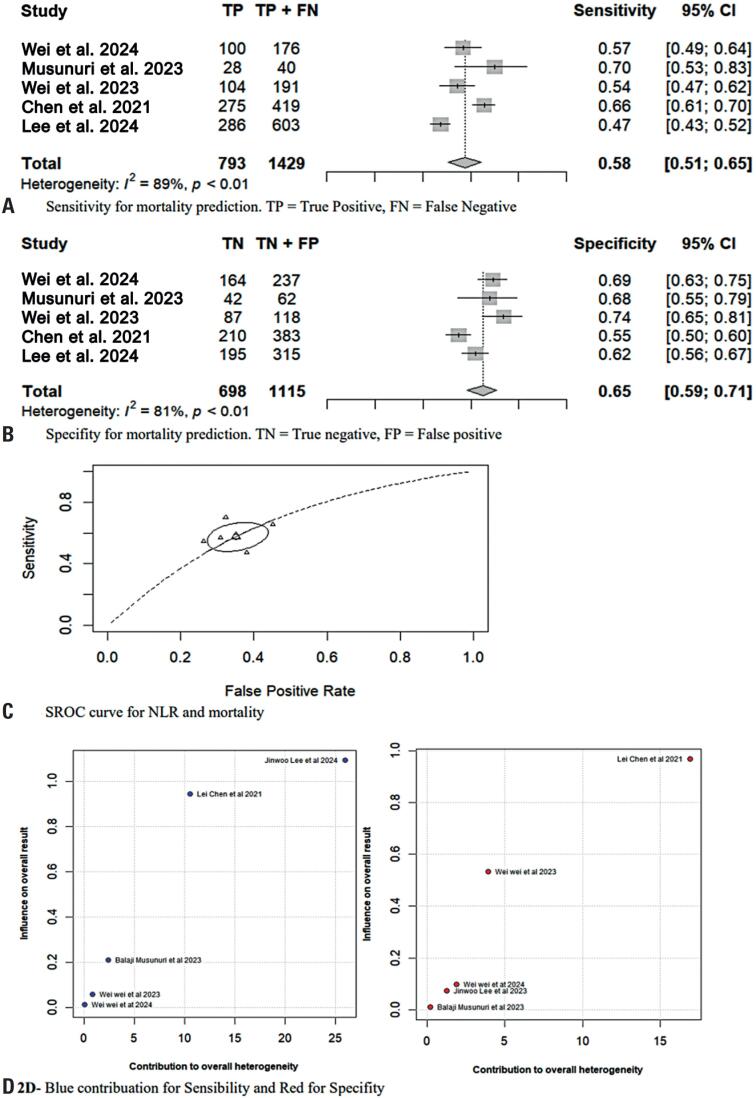
Sensitivity specificity SROC and Baujat plot.

The univariate analysis, that is, analysis just of the NLR value from each study showed a pooled sensitivity of 0.58 (95%CI=0.51–0.65, p<0.01) for predicting mortality, with substantial heterogeneity (I²=89%; Figure 2A). The pooled specificity was 0.65 (95%CI=0.59–0.71, p<0.01, I²=81%; Figure 2B). In the bivariate analysis, the area under the curve (AUC) reached 0.65, with an optimal balance between sensitivity and specificity of 0.58 and 0.65, respectively (Figure 2C). A threshold effect of −0.19 suggested a consistent trend between sensitivity and specificity across the included studies.

**Figure 3:**
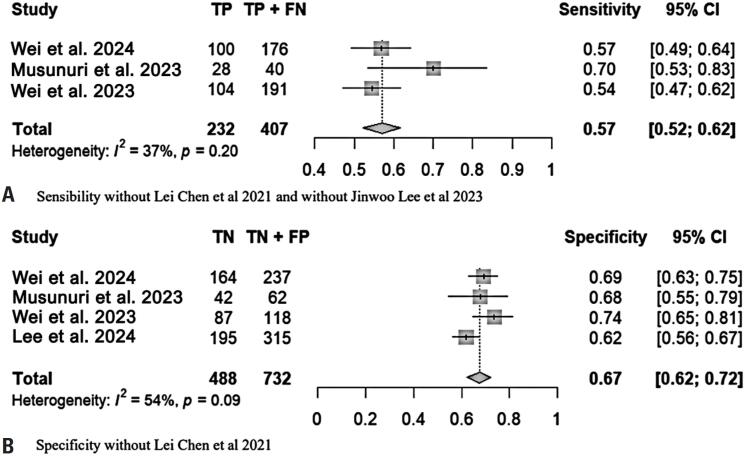
A) Statistical analysis excluding studies^(24)^ and^(25^); B) Statistical analysis excluding study^(25)^

TP: true positive; FN: false negative; TN true negative; FP: false positive.

To explore the heterogeneity of our meta-analysis, we chose to use the Baujat plot, as it provides a visual and straightforward representation of the quantity and influence of heterogeneity in each study on sensitivity and specificity (Figure 2D). Subsequently, we performed a sensitivity analysis by excluding the studies with the largest contributions (Figures 3A and 3B). As observed, two studies significantly contributed to the heterogeneity in sensitivity.^(24,25)^ For specificity, the contribution of one study was the most relevant.^(25)^ After excluding these studies, a new statistical evaluation revealed I²=37%, with a p-value of 0.20 for sensitivity, and I²=54%, with a p-value of 0.09 for specificity.

**Figure 4:**
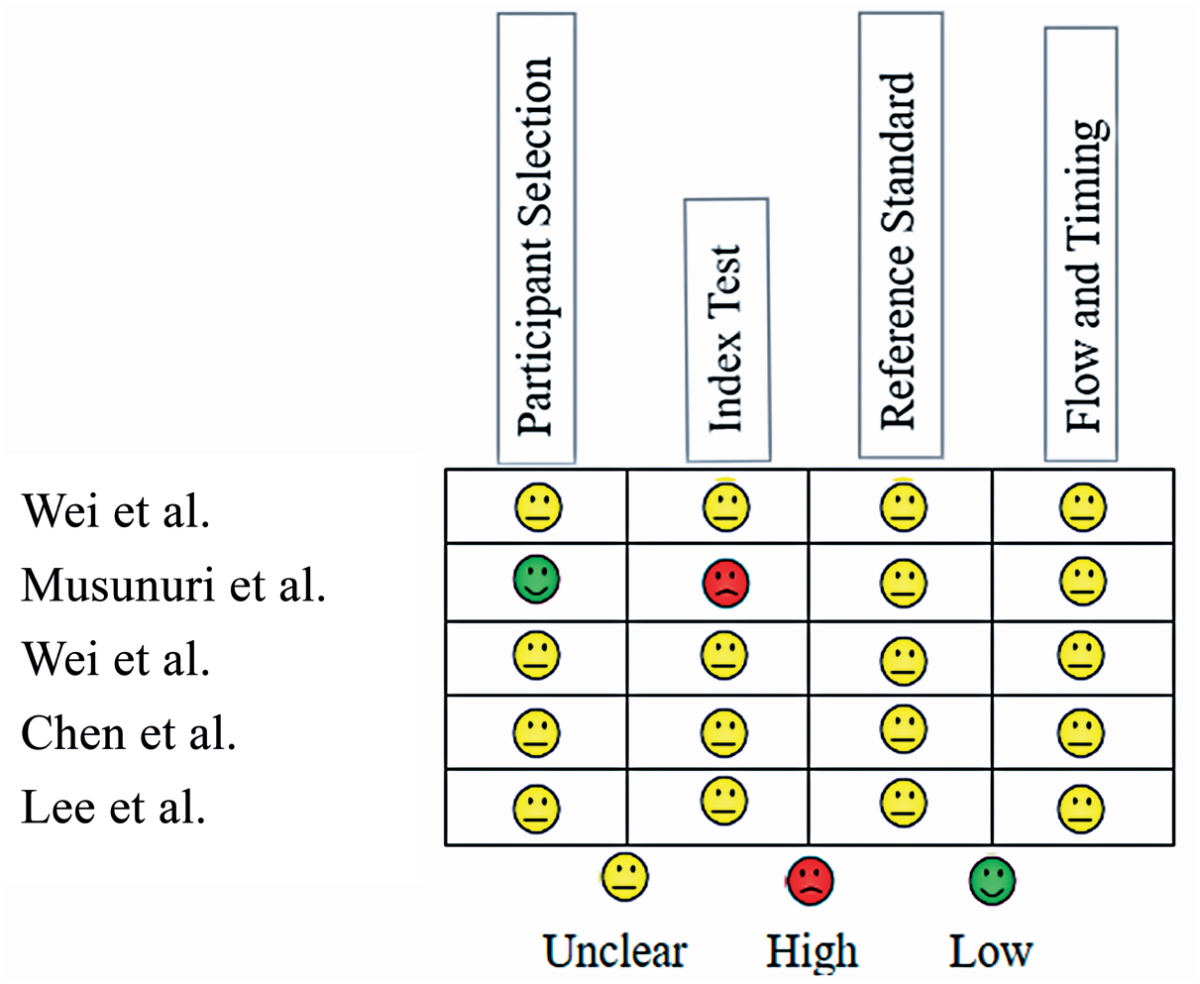
Assessing risk of bias of each study via QUADAS-2

We assessed the bias in each study using the QUADAS-2 tool (Figure 4). The study by Musunuri et al. exhibited the highest potential for bias^(22)^ as it did not establish a predetermined cutoff value for NLR and was thus classified as having a high risk of bias. Other studies were classified as having an indeterminate risk owing to a lack of clear data to answer the signaling questions.

## DISCUSSION

In this meta-analysis, we explored the utility of the NLR as a predictor of mortality in patients with AKI. Our findings suggest that an elevated NLR may play a role as a predictor of mortality in patients with AKI, which is consistent with the existing literature that points to the NLR as a relevant inflammatory marker in severe clinical conditions.

We observed significant variations in the NLR cut-off points between the studies. For example, one study used a dynamic approach to evaluate variations in the NLR between admission and clinical outcome,^(23)^ which may have influenced the sensitivity and specificity of their analyses. In addition, the possibility of patient overlap between the two studies^(23,26)^ may have affected the accuracy of the statistical results, raising questions regarding the robustness of the data.

Another highlight is the study,^(24)^ which presented results opposite to those expected, showing an inverse relationship between NLR and mortality in patients with septic AKI undergoing continuous renal replacement therapy. This discrepancy can be explained by the specific population analyzed, which included severely septic patients, in whom the inflammatory response and course of AKI differed from typical clinical scenarios. Despite the contradictory results, we retained the study in our analyses, as the aim was to evaluate the available data in the literature, not just what was the most convenient.

In addition, a prospective study showed a higher sensitivity (S=0.70) than other studies, suggesting that the NLR may be particularly useful for identifying patients at a higher risk of short-term mortality.^(22)^ This reinforces the potential of NLR as a tool for risk stratification, especially when considering its low cost and accessibility in clinical settings with limited resources.

We found that the sensitivity and specificity of NLR for predicting mortality in patients with AKI was 0.58, and the specificity was 0.65. Thus, this biomarker appears to perform better when used to predict a lower risk of death in patients with AKI.

A study evaluating TIMP-2 and IGFBP7 in high-risk surgical patients reported an AUC of 0.84 for predicting stage 2 or 3 AKI within 48 hours of admission.^(27)^ Furthermore, a meta-analysis of 10 studies involving 1,559 cases demonstrated a sensitivity of 0.82 (95% CI=0.77–0.86; I²=53.4%) and a specificity of 0.64 (95%CI=0.61–0.67; I²=88.3%) predicting mortality in patients with AKI.^(28)^ These AKI biomarkers, which reflect early cellular stress that precedes overt kidney damage, have consistently outperformed traditional markers such as serum creatinine and urine output in predicting AKI onset in specific patient populations.^(1,10,23,26)^ However, their ability to reliably predict mortality in patients with AKI remains an ongoing topic of discussion. Current evidence highlights the variability in their performance in predicting mortality across different settings and underscores the need for further research to validate their prognostic utility.

On the other hand, NLR, as an inflammatory marker, presented a lower AUC than TIMP-2 and IGFBP7. This comparison suggests that although the NLR is valuable owing to its simplicity and widespread accessibility, it may lack the sensitivity required to accurately predict mortality risk in critically ill patients with AKI. Thus, in this context, its role may be complementary, rather than primary.

## LIMITATIONS

Our study has several limitations. Among them, the significant heterogeneity of the meta-analysis may be attributed to differences in patient profiles across studies. Furthermore, owing to the methodology used, we did not establish a cut-off value for the NLR. Instead, we used the sensitivity/specificity provided by the primary studies to generate a 2 × 2 table and calculated true positives, true negatives, false positives, and false negatives. Without an NLR cutoff, its application becomes challenging, with the assistant physician responsible for selecting the most appropriate value for their practice or using the NLR suggested in the literature (*i.e*., an NLR of ∼ 2).^(5)^ Another limitation of our study was the design of the primary studies, the majority of which were retrospective, and the small number of studies included (n=5). The values of fewer than 10 studies made it difficult to assess publication bias using the funnel plot method, contributing to the study's limited strength. We attempted to minimize this issue using QUADAS-2.

## CONCLUSION

This study suggests that the neutrophil-to-lymphocyte ratio is associated with a poor prognosis in patients with acute kidney injury. These findings underscore the potential of neutrophil-to-lymphocyte ratio as a readily available prognostic marker in clinical practice, particularly in resource-limited settings. However, the significant heterogeneity between the included studies, particularly regarding cutoff values and patient populations, highlights the need for further prospective research with standardized methodologies to establish the definitive prognostic utility of neutrophil-to-lymphocyte ratio in acute kidney injury. Until then, neutrophil-to-lymphocyte ratio can serve as an adjunctive tool in risk stratification, especially in critically ill patients, but should be interpreted cautiously alongside other clinical parameters.

## Supplementary Materials

SUPPLEMENTARY MATERIAL Neutrophil-to-lymphocyte ratio as a predictor of mortality in patients with acute kidney injury a systematic review and meta-analysis
Idrys Henrique Leite Guedes, Fernando Uvinha, Isabela Lino Costa, Gustavo Martins de Ferreira, Luciano César Pontes de Azevedo, Paulo Ricardo Gessolo Lins
DOI: 10.31744/einstein_journal/2025RW1555
We conducted searches on PubMed, Embase, Cochrane Central Register of Controlled Trials (CENTRAL), and LILACS, with no date restriction. The complete search strategies for each database are provided below.
**Table 1S:** Search strategies

**PubMed** ("neutrophil-to-lymphocyte ratio"[tiab] OR "neutrophil to T-lymphocyte"[tiab] OR "neutrophil to lymphocyte ratio"[tiab] OR "neutrophil lymphocyte ratio"[tiab]) AND ("acute kidney injury"[MeSH] OR "kidney injury"[tiab] OR "renal injury"[tiab] OR "acute renal failure"[tiab] OR "kidney failure"[tiab])
**Embase** (‘neutrophil to lymphocyte ratio’/exp OR "neutrophil-to-lymphocyte ratio":ti, ab OR "neutrophil to T-lymphocyte":ti, ab OR "neutrophil to lymphocyte ratio":ti, ab OR "neutrophil lymphocyte ratio":ti, ab) AND (‘acute kidney injury’/exp OR "kidney injury":ti, ab OR "renal injury":ti, ab OR "acute renal failure":ti, ab OR "kidney failure":ti, ab)
**CENTRAL (Cochrane Central Register of Controlled Trials)** ("neutrophil-to-lymphocyte ratio" OR "neutrophil to T-lymphocyte" OR "neutrophil to lymphocyte ratio" OR "neutrophil lymphocyte ratio") AND ("acute kidney injury" OR "kidney injury" OR "renal injury" OR "acute renal failure" OR "kidney failure")
**LILACS** ((ti:("neutrophil-to-lymphocyte ratio" OR "neutrophil to T-lymphocyte" OR "neutrophil to lymphocyte ratio" OR "neutrophil lymphocyte ratio") OR ab:("neutrophil-to-lymphocyte ratio" OR "neutrophil to T-lymphocyte" OR "neutrophil to lymphocyte ratio" OR "neutrophil lymphocyte ratio"))) AND ((ti:("acute kidney injury" OR "kidney injury" OR "renal injury" OR "acute renal failure" OR "kidney failure") OR ab:("acute kidney injury" OR "kidney injury" OR "renal injury" OR "acute renal failure" OR "kidney failure")
